# Genomic Analysis of an IMP-1-Producing *Atlantibacter* *hermannii* Bloodstream Isolate Carrying a Transferable *bla*_IMP-1_-Harbouring IncHI2/pST1 Plasmid Related to Historical Enterobacterales Plasmids in Japan

**DOI:** 10.3390/microorganisms14102276

**Published:** 2026-10-06

**Authors:** Takashi Okanda, Natsuki Yumura, Daishu Fukushima, Niinyo Nakajima, Tetsuo Yamaguchi, Yosuke Tanaka, Kenta Katsumata, Kotaro Aoki, Takuro Koshikawa, Tadatomo Oyanagi, Tomonori Takano, Hiroyuki Kunishima, Mitsuo Kaku, Hiromu Takemura

**Affiliations:** 1 Department of Microbiology, St. Marianna University School of Medicine, 2-16-1 Sugao, Miyamae-ku, Kawasaki, Kanagawa 216-8511, Japan; takashi.okanda@marianna-u.ac.jp (T.O.);; 2 Department of Clinical Laboratory, St. Marianna University Yokohama Seibu Hospital, 1197-1 Yasashi-cho, Asahi-ku, Yokohama, Kanagawa 241-0811, Japan; 3 Department of Gastroenterological and General Surgery, St. Marianna University Yokohama Seibu Hospital, 1197-1 Yasashi-cho, Asahi-ku, Yokohama, Kanagawa 241-0811, Japan; 4 Department of Microbiology and Infectious Diseases, Toho University School of Medicine, 5-21-16 Omori-nishi, Ota-ku, Tokyo 143-8540, Japan; 5 Department of Clinical Laboratory Technology, St. Marianna University Hospital, 2-16-1 Sugao, Miyamae-ku, Kawasaki, Kanagawa 216-8511, Japan; 6 Department of Infectious Diseases, St. Marianna University School of Medicine, 2-16-1 Sugao, Miyamae-ku, Kawasaki, Kanagawa 216-8511, Japan

**Keywords:** *Atlantibacter hermannii*, *bla*_IMP-1_, IncHI2 plasmid, carbapenemase, integron, conjugation

## Abstract

*Atlantibacter hermannii* is an uncommon member of the Enterobacterales rarely associated with human infection, and genomic data on antimicrobial resistance in this species remain limited. Carbapenemase-producing *A. hermannii* isolates have rarely been described. We characterized an IMP-1-producing *A. hermannii* bloodstream isolate recovered in Japan and assessed the structure and transferability of its *bla*_IMP-1_-harbouring IncHI2 plasmid. Minimum inhibitory concentrations were determined by broth microdilution. Complete genome sequencing was performed using Oxford Nanopore technology, followed by comparative plasmid analysis and conjugation experiments. The isolate showed reduced susceptibility to carbapenems and resistance to extended-spectrum cephalosporins, whereas aztreonam, aztreonam–avibactam, and cefiderocol retained in vitro activity. Genome sequencing revealed a circular chromosome and two plasmids, including a 289-kb IncHI2 plasmid belonging to plasmid sequence type 1 (pST1) and harbouring *bla*_IMP-1_ within a class 1 integron. Comparative analysis showed structural conservation with IncHI2/pST1 plasmids identified in Japanese Enterobacterales isolates in 2007 and 2018. Conjugation experiments confirmed transfer to *Escherichia coli*, with markedly higher transfer frequencies at 30–35 °C than at 37 °C. These findings provide genomic and functional evidence for the continued detection of closely related *bla*_IMP-1_-harbouring IncHI2/pST1 plasmids among different Enterobacterales species in Japan over an extended period.

## 1. Introduction

Carbapenem-resistant Enterobacterales (CRE) represent a major global public health threat because of limited therapeutic options and the high mortality associated with severe infections. Among the various carbapenemases, IMP-type metallo-β-lactamases (MBLs) are widely disseminated in Japan and several other regions and are frequently associated with mobile genetic elements, particularly plasmids belonging to the IncHI2 incompatibility group [1,2].

*Atlantibacter hermannii*, formerly classified as *Escherichia hermannii*, is an opportunistic member of the Enterobacterales that is only rarely implicated in human infections [3]. Sporadic cases of bloodstream infection and device-associated infections have been reported [4,5,6], and a recent systematic review highlighted the limited number of documented human infections caused by this organism [7]. However, antimicrobial resistance mechanisms in this species remain poorly characterized. Carbapenemase production in *A. hermannii* has only rarely been described, including VIM-1- and IMP-4-producing isolates [8,9]. Although infections caused by this organism are uncommon, members of the Enterobacterales that are infrequently encountered in clinical settings may still participate in the carriage and dissemination of antimicrobial resistance genes through the acquisition of mobile genetic elements. Consequently, genomic data on carbapenemase-producing *A. hermannii* isolates are extremely limited, and complete genome characterization of IMP-producing strains has not been reported.

IncHI2 plasmids are recognized as important vehicles for the dissemination of antimicrobial resistance genes across Enterobacterales species. These plasmids frequently carry multiple resistance determinants embedded within integrons and resistance islands and can transfer efficiently between bacterial hosts [10,11,12]. Notably, IncHI plasmids often exhibit temperature-dependent conjugation properties that facilitate their spread under environmental and clinical conditions. Temperature-sensitive transfer has been well documented for IncHI plasmids such as R27, in which conjugative transfer is markedly reduced at 37 °C compared with lower temperatures [13].

In this study, we report the identification and complete genome characterization of an IMP-1–producing *A. hermannii* isolate recovered from a bloodstream infection in Japan. We further analyzed the structure and transferability of the *bla*_IMP-1_-harbouring IncHI2 plasmid and compared it with related plasmids identified in *Enterobacter hormaechei* isolated in Japan.

## 2. Materials and Methods

### 2.1. Bacterial Isolates

An *A. hermannii* SMU6841 isolate was recovered from blood cultures of a hospitalized patient at St. Marianna University School of Medicine Yokohama Seibu Hospital (Yokohama, Japan) in April 2025. Initial species identification was performed using matrix-assisted laser desorption ionization–time of flight mass spectrometry (MALDI-TOF MS) with the MALDI Biotyper system (Bruker Daltonics, Bremen, Germany). MALDI-TOF MS identified the isolate as *E. hermannii*, reflecting the former taxonomic designation used in the database. Antimicrobial susceptibility testing was initially performed using the MicroScan WalkAway system (Beckman Coulter, Brea, CA, USA) with dry plate panels (Eiken Chemical Co., Ltd., Tokyo, Japan), and minimum inhibitory concentrations (MICs) were interpreted according to Clinical and Laboratory Standards Institute (CLSI) guidelines. Carbapenemase production was evaluated using the modified carbapenem inactivation method (mCIM) and EDTA-modified carbapenem inactivation method (eCIM). The presence of *bla*_IMP_ was detected using the Xpert Carba-R assay (Cepheid, Sunnyvale, CA, USA) on the GeneXpert system according to the manufacturer’s instructions. The isolate was subsequently transferred to the Department of Microbiology, St. Marianna University School of Medicine for further genomic and microbiological analyses.

For comparative analysis, an IMP-1-producing *Enterobacter hormaechei* subsp. *hoffmannii* SMU6361 isolate harbouring an IncHI2 plasmid was included. This isolate was recovered at St. Marianna University School of Medicine Hospital (Kawasaki, Japan) in December 2018 and had been stored at −80 °C until use.

### 2.2. Antimicrobial Susceptibility Testing

MICs were determined by broth microdilution (BMD) according to CLSI guidelines [14]. Aztreonam–avibactam MICs were determined by BMD with avibactam at a fixed concentration of 4 μg/mL. The following antimicrobial agents were tested: ceftazidime, latamoxef, cefiderocol, aztreonam, imipenem, meropenem, piperacillin–tazobactam, ceftazidime–avibactam, aztreonam–avibactam, amikacin, minocycline, tigecycline, ciprofloxacin, trimethoprim–sulfamethoxazole, and colistin. Cefiderocol MICs were determined using BMD with iron-depleted cation-adjusted Mueller–Hinton broth (ID-CAMHB) according to CLSI recommendations [14]. Colistin MICs were determined using standard BMD. *Escherichia coli* ATCC 25922 was used as the quality control strain according to CLSI recommendations [14,15]. Results were interpreted according to CLSI M100 [15].

### 2.3. Whole-Genome Sequencing, Assembly, Polishing, and Validation

Whole-genome sequencing (WGS) was performed for *A. hermannii* SMU6841, *E. hormaechei* SMU6361, and two representative *Escherichia coli* ML4909 transconjugants, SMU6842 and SMU6845. For SMU6841, an independent second sequencing run was additionally performed to validate the original genome assembly. High-molecular-weight genomic DNA was extracted using the magLEAD system with the magDEA Dx SV and PS protocol (Precision System Science Co., Ltd., Chiba, Japan). Sequencing libraries were prepared using the Rapid Barcoding Kit 96 V14 (SQK-RBK114.96; Oxford Nanopore Technologies [ONT], Oxford, UK), and sequencing was performed on the MinION platform using R10.4.1 flow cells (FLO-MIN114; ONT).

Basecalling and demultiplexing were performed using Dorado version 1.1.1 with the dna_r10.4.1_e8.2_400bps_sup@v5.2.0 model. Reads were quality-filtered and adapter-trimmed using Fastplong version 0.4.1 (https://github.com/OpenGene/fastplong; accessed on 12 September 2026), followed by self-error correction using DeChat version 1.0.1 [16]. De novo assembly was performed using CLC Genomics Workbench version 26.0.2 (Qiagen, Aarhus, Denmark). The resulting assemblies were subjected to three successive rounds of polishing using the Polish Contigs with Reads tool in CLC Genomics Workbench version 26.0.2, using DeChat-corrected Oxford Nanopore reads. Genome annotation was performed using the DDBJ Fast Annotation and Submission Tool (DFAST, https://dfast.ddbj.nig.ac.jp/; accessed on 12 September 2026).

The two SMU6841 sequencing datasets were analyzed independently. Assembly reproducibility was assessed by comparing the number and sizes of reconstructed replicons, plasmid replicon type, plasmid sequence type (pST), resistance-gene organization, and whole-plasmid sequence similarity. Circularity of all reconstructed replicons was confirmed by long-read support across the respective circularization junctions. For pAH6841_IncHI2, long-read support was additionally examined across both flanking junctions of the *bla*_IMP-1_-containing class 1 integron. Sequencing and assembly quality metrics are summarized in Supplementary Table S1.

### 2.4. Genomic Analysis

Species identification was confirmed by average nucleotide identity (ANI) analysis [17]. ANI values were calculated using DFAST by comparing the assembled genome with publicly available *A. hermannii* reference genomes, including the type strain and representative genomes. Pairwise ANI values and alignment percentages (AP) were calculated using the Create Average Nucleotide Identity Comparison tool and visualized as a heatmap in CLC Main Workbench version 26.0.2. An ANI-based neighbor-joining tree was subsequently generated from the ANI comparison table using the Create Tree from Comparison function. CLC Main Workbench converts the ANI similarity matrix to a symmetric distance matrix before tree construction. No bootstrap or other branch-support analysis was performed. The tree was used for visualization of genome-based taxonomic placement and not for detailed phylogenetic inference.

Antimicrobial resistance genes, plasmid replicons, and pSTs were identified using ResFinder version 4.7.2, PlasmidFinder version 3.0.3, and pMLST version 2.0, respectively, through the Center for Genomic Epidemiology platform (https://www.genomicepidemiology.org/; accessed on 12 September 2026). Analyses were performed using the default settings of each tool. Integron structures and resistance gene arrangements were identified based on annotation results and manual inspection of genomic regions.

### 2.5. Plasmid Analysis

Plasmid analysis was performed to characterize the structure and transferability of the *bla*_IMP-1_-harbouring IncHI2 plasmids. For comparative analysis, we focused on complete *bla*_IMP-1_-harbouring IncHI2/pST1 plasmid sequences identified in Japan that were available for direct whole-plasmid structural comparison. Three plasmids meeting these criteria were included: the 2007 reference plasmid pMTY11043_IncHI2 (GenBank accession number AP018352.1), the 2018 plasmid pEH6361_IncHI2 (JBYUJE010000001.1), and the 2025 plasmid pAH6841_IncHI2 (JBXHAU010000002.1). Whole-plasmid comparisons and integron structure analyses were visualized using EasyFig version 3.0.0 to assess similarities in plasmid backbone structures and resistance gene organization. Pairwise whole-plasmid ANI and AP among the three IncHI2/pST1 plasmids were calculated using CLC Main Workbench as descriptive similarity metrics to support structural comparison. These metrics were not used as standalone criteria for plasmid lineage assignment.

### 2.6. Conjugation Experiments and Transconjugant Validation

Conjugation experiments were performed using the clinical isolates as donor strains and rifampicin-resistant *E. coli* ML4909 as the recipient strain. Donor suspensions were prepared at a McFarland No. 0.5, diluted 100-fold in Luria–Bertani (LB) broth, and incubated with shaking at 35 °C for 3 h to obtain approximately 1 × 10^8^ CFU/mL. The recipient suspension was adjusted to approximately 1 × 10^8^ CFU/mL. Donor and recipient suspensions (0.2 mL each) were mixed at a 1:1 ratio in a total mating volume of 0.4 mL and incubated without shaking at 30 °C, 35 °C, or 37 °C.

Samples were collected after 6 h and 24 h of mating and serially diluted as appropriate for colony enumeration. Donor, recipient, and transconjugant populations were enumerated on Mueller–Hinton agar containing latamoxef (1 μg/mL), rifampicin (64 μg/mL), and both rifampicin and latamoxef (64 and 1 μg/mL), respectively. Aliquots (100 μL) of the appropriate dilutions were plated and incubated overnight at 35 °C before colony counting. For transconjugant detection, 100 μL of the undiluted mating culture was also plated on the dual-selection medium. Transfer frequencies were calculated as the number of transconjugants per donor cell (T/D). The minimum detectable transconjugant density was therefore 10 CFU/mL. The detection limit for conjugation frequency was calculated for each experiment by dividing 10 CFU/mL by the corresponding donor density and ranged from 2.0 × 10^−10^ to 2.7 × 10^−8^ transconjugants per donor cell across all experimental conditions. Donor-only and recipient-only suspensions were plated on the same dual-selection medium as negative selection controls. No colonies were observed under these conditions, excluding background growth or spontaneous resistance at the detection level of the assay. All experiments were performed independently five times (*n* = 5), and median values were reported.

Representative transconjugants derived from SMU6841 and SMU6361, designated SMU6842 and SMU6845, respectively, were subjected to WGS. Acquisition of the donor IncHI2 plasmids was evaluated by comparing plasmid size, replicon type, pST, integron structure, antimicrobial resistance and accessory gene content, and whole-plasmid ANI and alignment percentage with those of the corresponding donor plasmids. Functional acquisition of antimicrobial resistance was further assessed by comparing meropenem and latamoxef MICs between the transconjugants and the recipient strain.

### 2.7. Statistical Analysis

Conjugation frequencies were log10-transformed before statistical analysis. For each plasmid, temperature (30, 35, and 37 °C) and mating time (6 and 24 h) were treated as two within-experiment factors in a two-way repeated-measures analysis of variance (ANOVA). Each independently performed conjugation experiment constituted one biological experimental unit (*n* = 5), and each experimental unit contributed matched observations to all six temperature × mating-time combinations. Greenhouse–Geisser correction was applied to the temperature main effect and the temperature × mating-time interaction to account for possible departures from sphericity. No sphericity correction was required for mating time because it comprised only two levels. Given the small number of biological replicates, formal normality testing was not considered sufficiently informative. The log10 transformation was used to reduce skewness in the conjugation-frequency data. Pairwise comparisons among temperatures at each mating time and between mating times at each temperature were performed using paired comparisons with Šídák correction for multiple testing. Statistical analyses were performed using Python version 3.13.5 with statsmodels version 0.14.6 and SciPy version 1.17.0. A two-sided *p* value < 0.05 was considered statistically significant.

## 3. Results

### 3.1. Clinical Isolate and Initial Characterization

The SMU6841 isolate was recovered from blood cultures obtained from a 54-year-old male patient who was emergently admitted on 15 April 2025, with abdominal distension. He was diagnosed with obstructive colitis associated with sigmoid diverticulitis and colonic stenosis. During hospitalization, the patient developed fever on April 28, and blood cultures subsequently yielded IMP-producing *A. hermannii* on May 1. Antimicrobial therapy with ciprofloxacin (400 mg every 8 h) plus metronidazole (500 mg every 8 h) was initiated on the same day. However, follow-up blood cultures remained positive on May 8, and aztreonam (2 g every 8 h) was added to the regimen. Blood cultures became negative on May 10. On May 16, the patient underwent colostomy for source control, and he subsequently recovered and was discharged on May 31. His medical history included mechanical aortic valve replacement and right total hip arthroplasty.

Initial species identification using MALDI-TOF MS identified the isolate as *E. hermannii* with a score of 2.30, reflecting the historical taxonomic designation retained in the MALDI-TOF MS database rather than uncertainty in species identification. Carbapenemase production was confirmed by positive results in both mCIM and eCIM, indicating MBL activity. The Xpert Carba-R assay detected *bla*_IMP_.

### 3.2. Antimicrobial Susceptibility Profile

MICs indicated resistance to extended-spectrum cephalosporins, latamoxef, and ceftazidime–avibactam. The isolate showed intermediate susceptibility to ciprofloxacin (MIC 0.5 µg/mL). The isolate remained susceptible to imipenem (MIC 1 µg/mL) but showed intermediate susceptibility to meropenem (MIC 2 µg/mL). In contrast, aztreonam retained activity (MIC ≤ 0.03 µg/mL), and both aztreonam–avibactam and cefiderocol exhibited potent in vitro activity. Detailed MIC values are shown in Table 1.

### 3.3. Genome Structure, Assembly Validation, and Species Confirmation

The original sequencing run of SMU6841 generated 122,676,639 bases of long-read data, corresponding to an approximate sequencing depth of 24×. De novo assembly yielded three circular replicons with a total length of 5,065,650 bp, consisting of a 4,685,000-bp chromosome, the 289,379-bp pAH6841_IncHI2 plasmid, and the 91,271-bp pAH6841.2 plasmid. The chromosome harboured the intrinsic resistance genes *bla*_HERA-5_ and *catA*. To independently validate the original assembly, SMU6841 was subjected to a second whole-genome sequencing run. The repeat sequencing generated 182,688,534 bases of long-read data, corresponding to an approximate sequencing depth of 36×. Independent de novo assembly reproduced the same three-circular-replicon genome architecture, consisting of a 4,684,500-bp chromosome, a 289,382-bp IncHI2/pST1 plasmid, and a 91,271-bp second plasmid. Whole-plasmid comparison of pAH6841_IncHI2 reconstructed from the two independent sequencing runs showed 100% ANI and 100% AP at the reported precision. Only three base-pair differences were identified in two non-annotated regions, consisting of a 2-bp insertion and a 1-bp insertion in the repeat assembly. No differences were observed in the *bla*_IMP-1_-containing class 1 integron or other annotated resistance-associated regions. The circularization junction of pAH6841_IncHI2 and both flanking junctions of the *bla*_IMP-1_-containing integron were independently supported by long reads from both sequencing datasets. Detailed sequencing and assembly quality metrics are provided in Supplementary Table S1.

**Figure 1:**
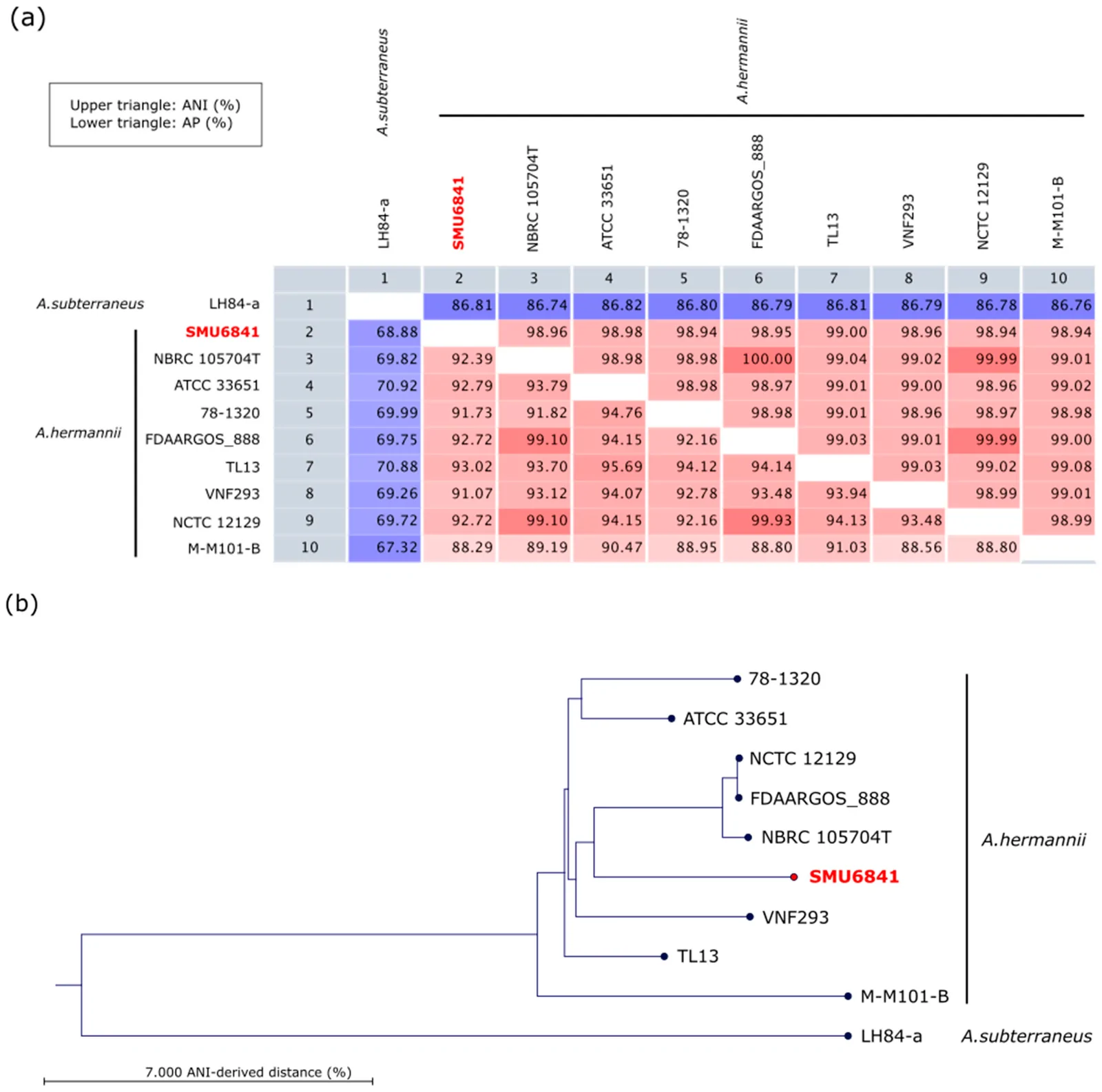
Genome-based taxonomic placement of SMU6841. (**a**) Heatmap showing pairwise average nucleotide identity (ANI; upper triangle) and alignment percentage (AP; lower triangle) among SMU6841 and representative *Atlantibacter hermannii* genomes. Cell colors represent the magnitude of ANI or AP values, with warmer colors indicating higher values and cooler colors indicating lower values. SMU6841 is highlighted in red in both panels. (**b**) ANI-based neighbor-joining tree generated from the ANI comparison table using CLC Main Workbench version 26.0.2 for visualization of genome-based taxonomic placement rather than detailed phylogenetic inference. No branch-support analysis was performed. SMU6841 clustered with the *A. hermannii* reference genomes. Reference genomes included *A. hermannii* FDAARGOS_888 (GCA_016027855.1), NBRC 105704T (GCA_000248015.2), ATCC 33561 (GCA_008064855.1), 78-1320 (GCA_011068345.1), TL13 (GCA_030166395.1), VNF293 (GCA_040369615.1), NCTC12129 (GCA_900635495.1), and M-M101-B (GCA_965182305.1). *Atlantibacter subterraneus* LH84-a (GCA_024224615.1) was included as an outgroup. Branch lengths represent ANI-derived distances expressed in percentage points.

ANI analysis against publicly available *A. hermannii* genomes demonstrated ANI values ranging from 98.9% to 99.0%, confirming the isolate as *A. hermannii*. The ANI-based neighbor-joining analysis placed SMU6841 within the *A. hermannii* cluster and was used for genome-based taxonomic visualization rather than detailed phylogenetic inference (Figure 1).

### 3.4. Plasmid Characterization

The *bla*_IMP-1_ was located on a 289,379-bp IncHI2 plasmid designated pAH6841_IncHI2 (Table 2). Plasmid multilocus sequence typing (pMLST) classified this plasmid as IncHI2 pST1. The *bla*_IMP-1_ was embedded within a class 1 integron comprising *intI1*, the *bla*_IMP-1_–*aac(6′)-IIc* gene cassette array, and the 3′ conserved region containing *qacEΔ1*–*sul1*. Additional antimicrobial resistance genes, including *tet(B)* and *qnrB6*, were also identified on the same plasmid. The plasmid additionally carried the tellurite resistance gene *terC*. A second plasmid, pAH6841.2 (91,271 bp), was untypeable and did not harbour carbapenemase or other major antimicrobial resistance genes.

For comparative analysis, an IncHI2 plasmid (pEH6361_IncHI2, 353,299 bp, pST1) identified in *E. hormaechei* also harboured *bla*_IMP-1_ within a class 1 integron with the same organization, comprising *intI1*, the *bla*_IMP-1_–*aac(6′)-IIc* gene cassette array, and the 3′ conserved region containing *qacEΔ1*–*sul1*. The plasmid also contained additional resistance genes, including *tet(B)*, *qnrB6*, and *terC* (Table 2).

### 3.5. Comparative Plasmid Analysis

**Figure 2:**
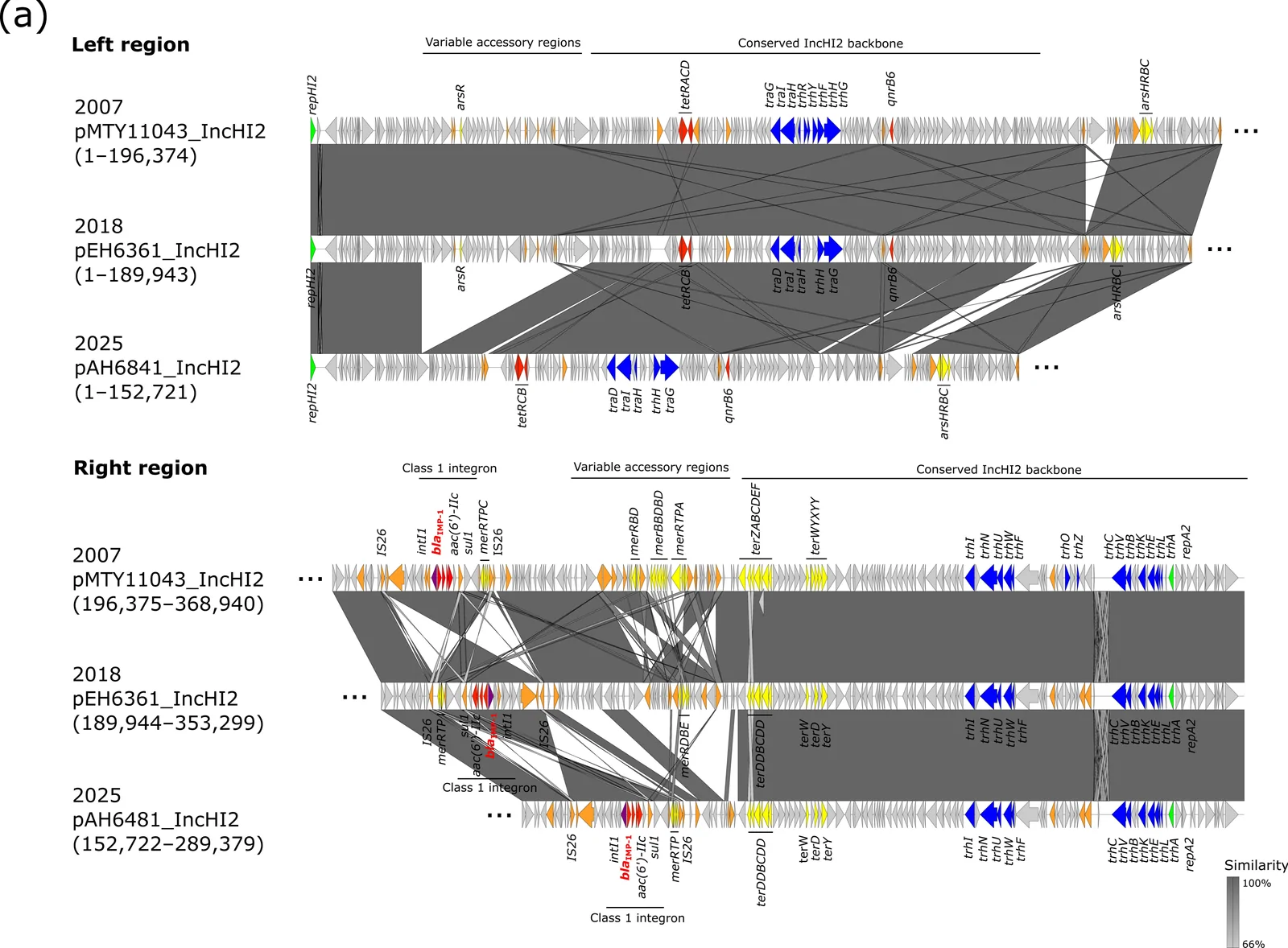

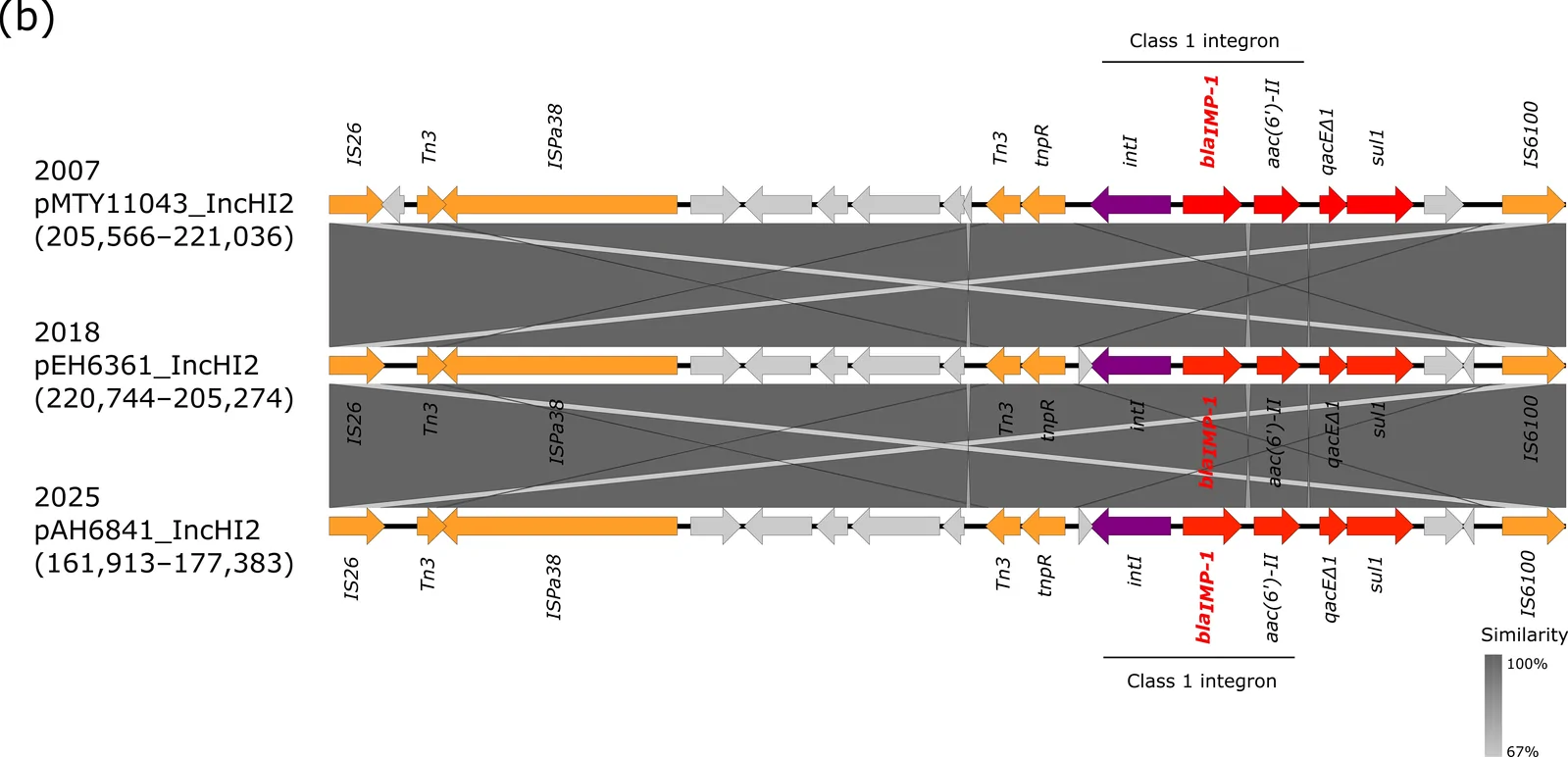
Structural comparison of three *bla*_IMP-1_-harbouring IncHI2 plasmids. Comparative analysis of *bla*_IMP-1_-harbouring IncHI2/pST1 plasmids identified in Japan in 2007 (pMTY11043_IncHI2), 2018 (pEH6361_IncHI2), and 2025 (pAH6841_IncHI2). (**a**) Whole-plasmid comparison of pMTY11043_IncHI2, pEH6361_IncHI2, and pAH6841_IncHI2 generated using EasyFig. Numbers in parentheses indicate nucleotide coordinates of the displayed regions, and ellipses indicate omitted intervening or flanking sequence. Extensive syntenic regions shared among the three plasmids are indicated as conserved IncHI2 backbone regions, whereas regions showing interrupted alignment and differences in gene content are indicated as variable accessory regions. For visualization purposes, the plasmid sequences are shown in two segments. (**b**) Detailed comparison of the class 1 integron regions showing the conserved organization of *intI1*, the *bla*_IMP-1_–*aac(6′)-IIc* gene cassette array, and the 3′ conserved region containing *qacEΔ1–sul1*. The integron regions were extracted from the upstream *IS26* to the downstream *IS6100* elements. The pEH6361_IncHI2 sequence was reversed for visualization to align the integron regions in the same orientation. Block arrows indicate confirmed or putative open reading frames (ORFs) and their orientations, with arrow length proportional to the predicted ORF size. The color scheme is as follows: green, replication genes; blue, conjugative transfer genes; orange, transposase genes; yellow, heavy-metal resistance genes; purple, integrase genes; red, antimicrobial resistance genes; gray, hypothetical or uncharacterized genes. Shaded regions indicate nucleotide similarity.

Comparative analysis of IncHI2 plasmids revealed substantial structural conservation among the plasmids identified in Japan, including pMTY11043_IncHI2 (2007), pEH6361_IncHI2 (2018), and pAH6841_IncHI2 (2025) (Figure 2a). All three plasmids belonged to IncHI2/pST1 and shared the same *bla*_IMP-1_-containing class 1 integron organization. These plasmids shared a highly conserved IncHI2 backbone structure despite differences in host species and isolation years. In particular, the class 1 integron consistently comprised *intI1*, the gene cassette array *bla*_IMP-1_–*aac(6′)-IIc*, and the 3′ conserved region *qacEΔ1–sul1* and was flanked by mobile genetic elements including *IS26* and *IS6100* (Figure 2b). The overall organization of the multidrug resistance region was largely conserved among these plasmids, although minor rearrangements in surrounding insertion sequences were observed. Pairwise whole-plasmid ANI values ranged from 99.89% to 99.99%, whereas alignment percentages ranged from 86.91% to 97.39% (Supplementary Table S2). Comparisons involving pAH6841_IncHI2 showed high nucleotide identity within aligned regions (ANI, 99.89–99.90%) but lower overall alignment percentages (86.91–87.82%), indicating reduced shared sequence fractions consistent with variation in accessory regions. ANI and AP were used as descriptive similarity metrics and not as standalone criteria for plasmid lineage assignment.

### 3.6. Plasmid Transfer and Functional Confirmation

**Figure 3:**
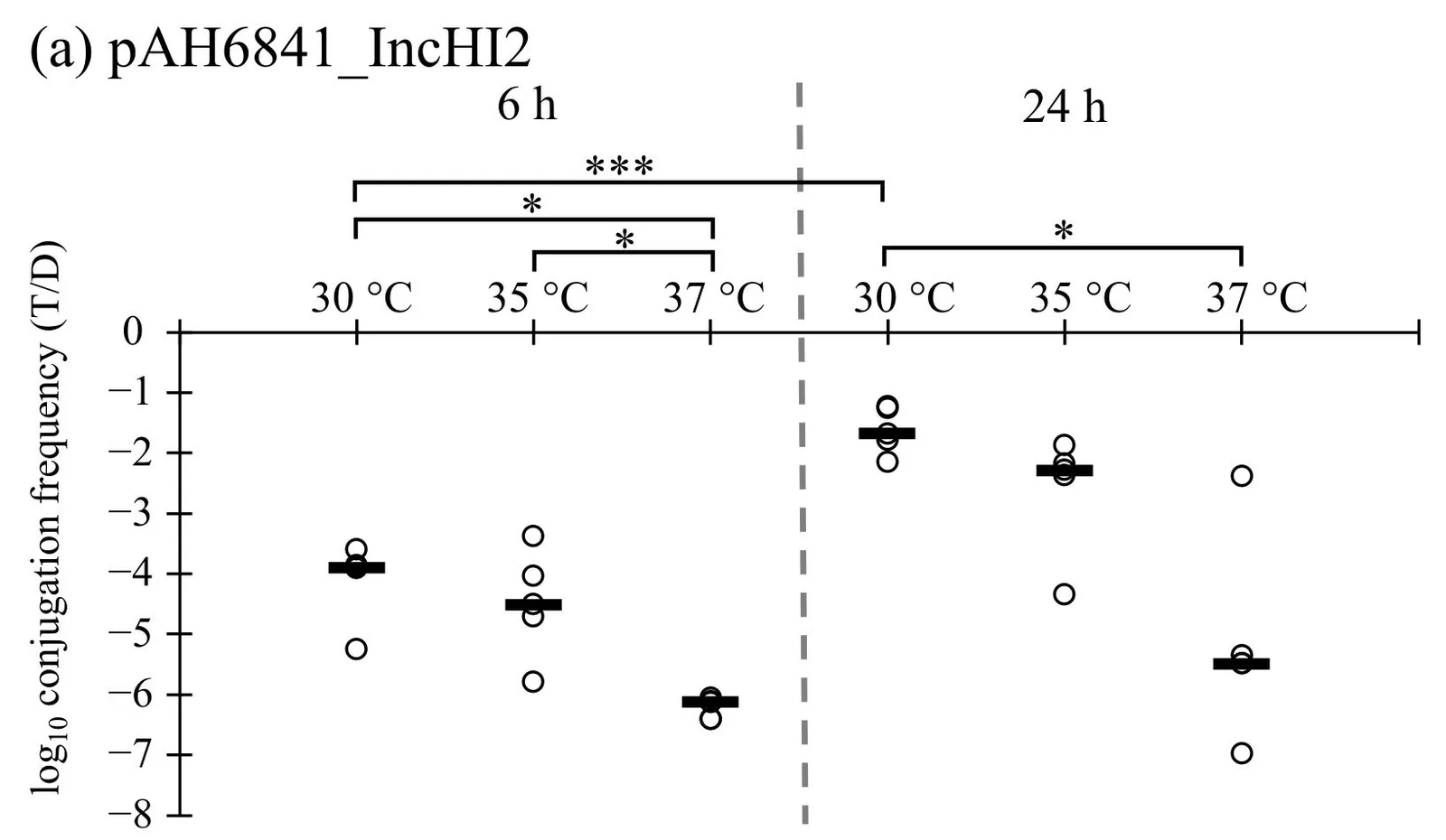

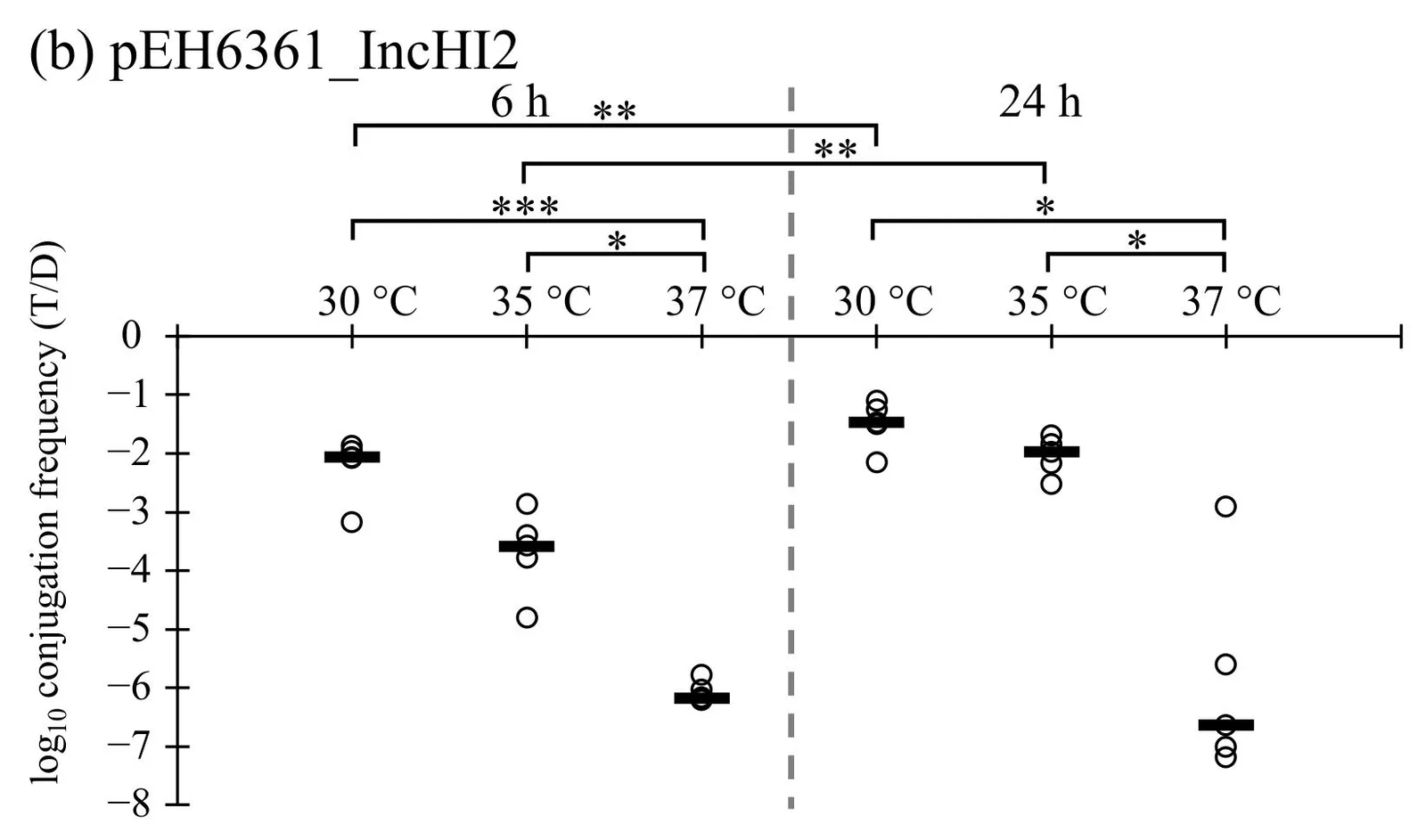
Temperature-dependent conjugation frequencies of IncHI2 plasmids. Conjugation frequencies of pAH6841_IncHI2 (**a**) and pEH6361_IncHI2 (**b**) were determined at 30 °C, 35 °C, and 37 °C after 6 h and 24 h of mating. Conjugation frequency was calculated as the number of transconjugants per donor cell (T/D). Data represent five independent biological experiments (*n* = 5) and are shown as individual values with median bars. The *y*-axis shows log10-transformed conjugation frequencies. The vertical dashed line separates the 6 h and 24 h mating conditions. Statistical comparisons were performed using two-way repeated-measures ANOVA, with Greenhouse–Geisser correction applied to effects involving temperature, followed by paired comparisons with Šídák correction for multiple testing. Brackets indicate statistically significant pairwise comparisons. * *p* < 0.05, ** *p* < 0.01, and *** *p* < 0.001. Exact Šídák-adjusted *p* values are reported in Section 3 Results.

Conjugation experiments demonstrated transfer of the *bla*_IMP-1_-harbouring IncHI2 plasmids to rifampicin-resistant *E. coli* ML4909. Transfer frequencies (T/D) showed marked temperature dependence for both plasmids (Figure 3). For pAH6841_IncHI2, median transfer frequencies (interquartile range [IQR]) at 6 h were 1.3 × 10^−4^ (1.3 × 10^−4^–1.4 × 10^−4^), 3.2 × 10^−5^ (2.0 × 10^−5^–9.5 × 10^−5^), and 7.7 × 10^−7^ (4.0 × 10^−7^–7.9 × 10^−7^) at 30 °C, 35 °C, and 37 °C, respectively. At 24 h, the corresponding values were 2.1 × 10^−2^ (1.7 × 10^−2^–5.6 × 10^−2^), 5.3 × 10^−3^ (4.4 × 10^−3^–6.8 × 10^−3^), and 3.3 × 10^−6^ (3.3 × 10^−6^–4.6 × 10^−6^), respectively. For pEH6361_IncHI2, median transfer frequencies (IQR) at 6 h were 8.8 × 10^−3^ (8.4 × 10^−3^–1.1 × 10^−2^), 2.7 × 10^−4^ (1.7 × 10^−4^–4.0 × 10^−4^), and 6.8 × 10^−7^ (6.5 × 10^−7^–9.4 × 10^−7^) at 30 °C, 35 °C, and 37 °C, respectively. At 24 h, the corresponding values were 3.5 × 10^−2^ (3.2 × 10^−2^–5.7 × 10^−2^), 1.1 × 10^−2^ (7.0 × 10^−3^–1.4 × 10^−2^), and 2.4 × 10^−7^ (9.9 × 10^−8^–2.6 × 10^−6^), respectively.

Two-way repeated-measures ANOVA of log10-transformed conjugation frequencies, with Greenhouse–Geisser correction for effects involving temperature, demonstrated significant effects of temperature and mating time for both plasmids. For pAH6841_IncHI2, significant effects were observed for temperature [F(1.25, 5.01) = 39.64, *p* = 0.00122] and mating time [F(1, 4) = 46.76, *p* = 0.00239], whereas the temperature × mating-time interaction was not significant [F(1.68, 6.73) = 1.47, *p* = 0.290]. For pEH6361_IncHI2, significant effects were similarly observed for temperature [F(1.82, 7.30) = 99.95, *p* = 5.77 × 10^−6^] and mating time [F(1, 4) = 20.28, *p* = 0.0108], whereas the interaction was not significant [F(1.06, 4.23) = 1.74, *p* = 0.256]. For pAH6841_IncHI2, transfer frequencies at 37 °C were significantly lower than those at 30 °C at both 6 h (Šídák-adjusted *p* = 0.0188) and 24 h (*p* = 0.0395), and lower than those at 35 °C at 6 h (*p* = 0.0486). Transfer frequency increased significantly from 6 h to 24 h at 30 °C (*p* = 0.00045). For pEH6361_IncHI2, transfer frequencies at 37 °C were significantly lower than those at 30 °C at both 6 h (*p* = 0.00043) and 24 h (*p* = 0.0273), and lower than those at 35 °C at both 6 h (*p* = 0.0200) and 24 h (*p* = 0.0347). Transfer frequencies increased significantly from 6 h to 24 h at 30 °C (*p* = 0.0082) and 35 °C (*p* = 0.0037).

WGS of representative transconjugants confirmed acquisition of circular IncHI2/pST1 plasmids structurally corresponding to the donor plasmids. SMU6842 harboured the 289,382-bp pTC6841_IncHI2 plasmid, whereas SMU6845 harboured the 353,300-bp pTC6361_IncHI2 plasmid. Both transconjugant plasmids retained the *bla*_IMP-1_-containing class 1 integron and the additional resistance/accessory genes identified in their corresponding donor plasmids. Whole-plasmid comparisons showed 100% ANI and 100% alignment percentage between each transconjugant plasmid and its corresponding donor plasmid (Table 2). These genomic findings supported transfer of the donor IncHI2/pST1 plasmids into the recipient strain. Antimicrobial susceptibility testing further demonstrated increased meropenem and latamoxef MICs in the transconjugants compared with *E. coli* ML4909 (Table 3).

## 4. Discussion

This study provides a comprehensive genomic characterization of an IMP-1-producing *A. hermannii* bloodstream isolate and demonstrates that this uncommon species can harbour a transferable *bla*_IMP-1_-containing IncHI2/pST1 plasmid. Although *A. hermannii* is an uncommon human pathogen, previous reports have demonstrated its involvement in bloodstream and device-associated infections [4,5,6,7], indicating that even infrequently encountered Enterobacterales species can harbour clinically important mobile resistance determinants.

The isolate exhibited a resistance profile consistent with MBL production, while aztreonam and cefiderocol retained in vitro activity. Notably, despite harbouring *bla*_IMP-1_, the isolate remained susceptible to imipenem (MIC, 1 μg/mL) and showed intermediate susceptibility to meropenem (MIC, 2 μg/mL). Low carbapenem MICs have been reported among IMP-producing Enterobacterales, indicating that carbapenemase carriage does not invariably result in phenotypic carbapenem resistance [18]. The specific factors underlying the relatively low carbapenem MICs in SMU6841 were not investigated. The observed cefiderocol activity was consistent with previous findings in multidrug-resistant Gram-negative isolates [19]. The isolate showed intermediate susceptibility to ciprofloxacin, which was included in the initial antimicrobial regimen. Blood cultures remained positive during the initial antimicrobial therapy but became negative two days after aztreonam was added. The temporal association between aztreonam administration and clearance of bacteremia was consistent with the observed in vitro susceptibility. However, because other antimicrobial agents were administered concomitantly, the clearance of bacteremia cannot be attributed solely to aztreonam. Subsequent surgical source control also contributed to the patient’s overall clinical recovery.

Genomic analysis demonstrated that *bla*_IMP-1_ was located on a large IncHI2 plasmid belonging to pST1 and embedded within a class 1 integron structure. IncHI2 plasmids are recognized as major vehicles for the dissemination of antimicrobial resistance genes among Enterobacterales [10,11,12], and class 1 integrons represent conserved platforms facilitating the mobilization of resistance determinants [20]. The conserved organization of the *bla*_IMP-1_-containing class 1 integron among the plasmids compared in this study indicates structural conservation of this resistance-associated region but does not by itself establish its persistence or contribution to dissemination.

Comparative plasmid analysis using EasyFig demonstrated substantial conservation of the IncHI2 backbone and the *bla*_IMP-1_-containing class 1 integron among the plasmids identified in 2007, 2018, and 2025. Pairwise ANI values were uniformly high within aligned regions, whereas the lower alignment percentages observed in comparisons involving pAH6841_IncHI2 reflected differences in the overall shared sequence fraction, particularly in accessory regions. These ANI and AP values were interpreted as descriptive measures of sequence similarity and were not considered evidence of direct evolutionary descent or continuity of a single plasmid lineage. A previous study reported that the Japanese plasmid pMTY11043_IncHI2 exhibits structural similarity to related IncHI2 plasmids identified in Taiwan and Australia [2], suggesting that these plasmids are part of a broader IncHI2 plasmid background associated with carbapenemase dissemination. In the present study, the plasmids identified in 2018 and 2025 showed strong structural similarity to this reference plasmid. These observations provide evidence that related *bla*_IMP-1_-harbouring IncHI2/pST1 plasmids have remained detectable in different Enterobacterales species in Japan over an extended period. Although the overall backbone and *bla*_IMP-1_-containing integron were conserved, differences were observed mainly in accessory regions, consistent with variation in the overall plasmid structure.

Conjugation experiments demonstrated efficient plasmid transfer to *E. coli*, with marked temperature dependence. Transfer frequencies were substantially higher at 30–35 °C than at 37 °C, consistent with previous reports on IncHI plasmids such as R27 [13] and with studies describing the regulatory mechanisms underlying temperature-dependent conjugative transfer in IncHI plasmids [21]. The observed temperature dependence suggests that environmental conditions may influence the transfer efficiency of these plasmids. However, the present experiments do not provide direct evidence of environmental dissemination or long-term persistence in natural settings.

This study has several limitations. First, the findings are based on a single clinical isolate, and therefore the prevalence of carbapenemase-producing *A. hermannii* remains unknown. Second, although comparative plasmid analysis demonstrated the detection of structurally related IncHI2/pST1 plasmids at different time points, the limited dataset does not establish continuous plasmid persistence, transmission, or direct evolutionary continuity. The plasmid comparison was intentionally restricted to complete *bla*_IMP-1_-harbouring IncHI2/pST1 plasmid sequences identified in Japan to allow a focused comparison under consistent geographic and plasmid-genetic criteria. Consequently, related IncHI2 plasmids identified outside Japan or carrying other IMP variants were not included in the direct structural comparison. In addition, only a limited number of complete *bla*_IMP-1_-harbouring IncHI2 plasmid sequences from Japan were available for comparison. Further genomic surveillance is required to clarify the epidemiology and dissemination dynamics of *bla*_IMP-1_-harbouring plasmids.

In conclusion, we identified an IMP-1-producing *A. hermannii* bloodstream isolate harbouring a transferable IncHI2/pST1 plasmid. Comparative genomic analysis demonstrated that this plasmid was structurally related to *bla*_IMP-1_-harbouring IncHI2/pST1 plasmids previously identified in different Enterobacterales species in Japan in 2007 and 2018. These findings document the continued detection of closely related resistance plasmids across different host species over an extended period and highlight the ability of uncommon Enterobacterales species to harbour clinically important mobile resistance elements.

## Figures and Tables

**Table 1:** Antimicrobial susceptibility of the *Atlantibacter hermannii* clinical isolate.

Antimicrobial Agent	MIC (μg/mL)	CLSI
Ceftazidime	>32	R
Latamoxef	>32	R
Cefiderocol	0.5	S
Aztreonam	≤0.03	S
Imipenem	1	S
Meropenem	2	I
Piperacillin–tazobactam	8/4	S
Ceftazidime–avibactam	>32/4	R
Aztreonam–avibactam	≤0.015/4	S
Amikacin	≤4	S
Minocycline	8	I
Tigecycline	0.5	N/D
Ciprofloxacin	0.5	I
Trimethoprim–sulfamethoxazole	≤0.5/9.5	S
Colistin	≤0.5	I

MICs were determined by broth microdilution and interpreted according to CLSI M100 (36th edition). Tigecycline breakpoint not defined (N/D). Colistin was interpreted according to CLSI M100 criteria for Enterobacterales, for which no susceptible category is defined (intermediate, ≤2 μg/mL; resistant, ≥4 μg/mL).

**Table 2:** Genomic features of *bla*_IMP-1_-harbouring IncHI2 plasmids identified in donor isolates and representative transconjugants.

Donor /TC	Strain ID	Species	Plasmid	Size (bp)	Inc Type	pMLST	Circular	Integron Structure	Additional AMR /Accessory Genes	ANI vs. Donor (%)	AP vs. Donor (%)
Donor	SMU6841	*A. hermannii*	pAH6841_IncHI2	289,379	IncHI2	pST1	Yes	*intI1*–*bla*_IMP-1_–*aac(6′)-IIc*–*qacEΔ1*–*sul1*	*tet(B)*, *qnrB6*, *terC*	—	—
Donor	SMU6361	*E. hormaechei*	pEH6361_IncHI2	353,299	IncHI2	pST1	Yes	*intI1*–*bla*_IMP-1_–*aac(6′)-IIc*–*qacEΔ1*–*sul1*	*tet(B)*, *qnrB6*, *terC*	—	—
TC	SMU6842	*E. coli* ML4909	pTC6841_IncHI2	289,382	IncHI2	pST1	Yes	*intI1*–*bla*_IMP-1_–*aac(6′)-IIc*–*qacEΔ1*–*sul1*	*tet(B)*, *qnrB6*, *terC*	100%	100%
TC	SMU6845	*E. coli* ML4909	pTC6361_IncHI2	353,300	IncHI2	pST1	Yes	*intI1*–*bla*_IMP-1_–*aac(6′)-IIc*–*qacEΔ1*–*sul1*	*tet(B)*, *qnrB6*, *terC*	100%	100%

ANI and AP values for transconjugant plasmids were calculated relative to their corresponding donor plasmids. Whole-plasmid comparisons revealed no structural rearrangements affecting the IncHI2 backbone or *bla*_IMP-1_-containing integron.

**Table 3:** Antimicrobial susceptibility profiles of donor, recipient, and transconjugant strains.

Antimicrobial Agent	Recipient	Transconjugant (30 °C)		Donor	
	*E. coli* ML4909	SMU6842 (pTC6841_IncHI2)	SMU6845 (pTC6361_IncHI2)	*A. hermannii* (pAH6841_IncHI2)	*E. hormaechei* (pEH6361_IncHI2)
Meropenem	0.03	4	4	2	4
Latamoxef	0.12	>32	>32	>32	>32
Ceftazidime	0.25	>32	>32	>32	>32
Aztreonam	≤0.03	≤0.03	≤0.03	≤0.03	16
Aztreonam-avibactam	≤0.015/4	≤0.015/4	≤0.015/4	≤0.015/4	0.5/4
Rifampicin	>1024	>1024	>1024	4	16

MICs are expressed in μg/mL. For aztreonam–avibactam, avibactam was tested at a fixed concentration of 4 μg/mL, and MIC values are expressed as aztreonam/avibactam. Transconjugants were selected on Mueller–Hinton agar supplemented with rifampicin (64 μg/mL) and latamoxef (1 μg/mL). The recipient strain (*E. coli* ML4909) is rifampicin resistant. Antimicrobial susceptibility testing was performed according to CLSI guidelines.

## Data Availability

The assembled genome sequence of *A. hermannii* SMU6841 has been deposited in GenBank under accession numbers JBXHAU010000003.1 (chromosome), JBXHAU010000002.1 (pAH6841_IncHI2), and JBXHAU010000001.1 (pAH6841.2). The assembled genome sequence of *E. hormaechei* SMU6361 used for comparative analysis has been deposited in GenBank under accession numbers JBYUJE010000002.1 (chromosome) and JBYUJE010000001.1 (pEH6361_IncHI2). The raw Oxford Nanopore sequencing reads for the original sequencing run of SMU6841 and for SMU6361 have been deposited in the NCBI Sequence Read Archive (SRA) under accession numbers SRR38183117 and SRR38946000, respectively. Raw sequencing reads generated from the independent repeat sequencing of SMU6841 and from the representative transconjugants SMU6842 and SMU6845 have also been deposited in the SRA under accession numbers SRR40735113, SRR40745917, and SRR40707661, respectively. The sequence data are associated with BioProject accession numbers PRJNA1445562 and PRJNA1473392.

## Supplementary Materials

The following supporting information can be downloaded at https://www.mdpi.com/article/10.3390/microorganisms14102276/s1, Table S1: Sequencing and assembly quality metrics of isolates and representative transconjugants; Table S2: Pairwise sequence similarity metrics among *bla*_IMP-1_-harbouring IncHI2/pST1 plasmids identified in Japan.
