# Supplementary material for: Genomic Analysis of an IMP-1-Producing Atlantibacter hermannii Bloodstream Isolate Carrying a Transferable blaIMP-1-Harbouring IncHI2/pST1 Plasmid Related to Historical Enterobacterales Plasmids in Japan
Source: Microorganisms. 2026 Oct 6;14(10):2276. doi: 10.3390/microorganisms14102276 (PMC13707398; doi:10.3390/microorganisms14102276)
Supplement: Supplementary file 1 [file microorganisms-14-02276-s001.zip › microorganisms-4503903-supplementary.pdf]

Supplementary Table S1. Sequencing and assembly quality metrics of isolates and representative transconjugants.

Dataset	Raw reads	Raw bases	Raw read N50 (bp)	Approx. raw sequencing depth (x)	Final assembly size (bp)	No. of final replicons	Circular replicons	Final replicons sizes (bp)	SRA accession
SMU6841 Run 1	24,079	122,676,639	5,094	24	5,065,650	3	3/3	Chr: 4,685,000; P1: 289,379; P2: 91,271	SRR38183117
SMU6841 Run 2	22,743	182,688,534	8,033	36	5,065,153	3	3/3	Chr: 4,684,500; P1: 289,382; P2: 91,271	SRR40735113
SMU6361	137,608	1,020,914,922	24,855	199	5,123,709	2	2/2	Chr: 4,770,410; P1: 353,299	SRR38946000
SMU6842	41,075	312,395,937	7,606	64	4,890,071	2	2/2	Chr: 4,600,689; P1: 289,382	SRR40745917
SMU6845	61,466	470,193,955	7,650	95	4,953,990	2	2/2	Chr: 4,600,690; P1: 353,300	SRR40707661

Raw reads, raw bases, and raw read N50 were calculated from the demultiplexed Oxford Nanopore sequencing data before read filtering and error correction. Approximate raw sequencing depth was calculated by dividing the total number of raw bases by the size of the final polished assembly. Assembly size, number of replicons, and replicon sizes were determined from the final assemblies after three rounds of polishing. Chr, chromosome; P1 and P2, plasmids 1 and 2, respectively.

Supplementary Table S2. Pairwise sequence similarity metrics among *bla*$_{IMP-1}$-harbouring IncHI2/pST1 plasmids identified in Japan

Plasmid 1	Size (bp)	Plasmid 2	Size (bp)	ANI (%)	AP (%)
pMTY11043_IncHI2	368,940	pEH6361_IncHI2	353,299	99.99	97.39
pMTY11043_IncHI2	368,940	pAH6841_IncHI2	289,379	99.9	87.82
pEH6361_IncHI2	353,299	pAH6841_IncHI2	289,379	99.89	86.91

ANI, average nucleotide identity; AP, alignment percentage. ANI and AP were used as descriptive measures of nucleotide similarity within aligned regions and the overall aligned fraction, respectively. These metrics were not used as standalone criteria for plasmid lineage assignment or direct evolutionary descent.
